# A novel path-specific effect statistic for identifying the differential specific paths in systems epidemiology

**DOI:** 10.1186/s12863-020-00876-w

**Published:** 2020-08-08

**Authors:** Hongkai Li, Zhi Geng, Xiaoru Sun, Yuanyuan Yu, Fuzhong Xue

**Affiliations:** 1 Institute for Medical Dataology, Cheeloo College of Medicine, Shandong University, Jinan, 250000 People’s Republic of China; 2 Department of Biostatistics, School of Public Health, Cheeloo College of Medicine, Shandong University, Jinan, People’s Republic of China; 3 School of Mathematical Sciences, Peking University, Beijing, 100000 People’s Republic of China

**Keywords:** Causal diagram model, Causal inference, Identification, Path-specific effect

## Abstract

**Background:**

Biological pathways play an important role in the occurrence, development and recovery of complex diseases, such as cancers, which are multifactorial complex diseases that are generally caused by mutation of multiple genes or dysregulation of pathways.

**Results:**

We propose a path-specific effect statistic (PSE) to detect the differential specific paths under two conditions (e.g. case VS. control groups, exposure Vs. nonexposure groups). In observational studies, the path-specific effect can be obtained by separately calculating the average causal effect of each directed edge through adjusting for the parent nodes of nodes in the specific path and multiplying them under each condition. Theoretical proofs and a series of simulations are conducted to validate the path-specific effect statistic. Applications are also performed to evaluate its practical performances. A series of simulation studies show that the Type I error rates of PSE with Permutation tests are more stable at the nominal level 0.05 and can accurately detect the differential specific paths when comparing with other methods. Specifically, the power reveals an increasing trends with the enlargement of path-specific effects and its effect differences under two conditions. Besides, the power of PSE is robust to the variation of parent or child node of the nodes on specific paths. Application to real data of Glioblastoma Multiforme (GBM), we successfully identified 14 positive specific pathways in mTOR pathway contributing to survival time of patients with GBM. All codes for automatic searching specific paths linking two continuous variables and adjusting set as well as PSE statistic can be found in supplementary materials.

**Conclusion:**

The proposed PSE statistic can accurately detect the differential specific pathways contributing to complex disease and thus potentially provides new insights and ways to unlock the black box of disease mechanisms.

## Background

Biological pathways play a key role in the occurrence, development and recovery of complex diseases, such as cancers, which are multifactorial complex diseases that are generally caused by mutation of multiple genes or dysregulation of pathways [1]. Besides biological pathways, improving clinical treatment, and discovering drug targets and biomarkers, are a series of actions among molecules (including genes and protein, etc.) in a cell that lead to a certain product or a change in the cell [2–6]. Recently, with the still-ongoing development of high-throughput sequencing technology, the price is obviously falling, large numbers of related-pathway omics data are exponentially growing, thus it has become one of the most important resource to analyze biological pathways via high-throughput omics data [7]. During the past 10 years, several pathway knowledge databases have been built, such as KEGG, BioCyc, MetaCyc, Reactome, RegulonDB and PantherDB [8–13]. The establishment of these knowledge databases laid the foundation to study pathways contributing to the occurrence, development and recovery of complex diseases [14]. Pathway-related knowledge databases and omics data contain a wealth of disease-related knowledge and information, such as information on the related-pathway genes, molecular interactions in the same pathway, topology structure of pathways, gene expression, and so on. However, how to reveal the mechanism of biological regulation (e.g. SNP, gene and protein) on complex disease from observational pathway-related omics databases has become a great challenge.

Recently, some pathway analytical methods have been proposed to study human physiology, systems biology and modern drug development that provided the computational framework for data pathway analysis and biomarker selection [11–17]. These methods include functional enrichment analysis or gene set analysis (GSA) [14], pathway analysis within a Bayesian Network framework [15], pathway analysis approaches based on the adaptive rank truncated product statistic [16] and a sub-pathway-based approach to studying the joint effects of multiple genetic variants [17]. Although, these methods are suitable for omics data analysis in systems epidemiology, most of them fail to take into account the correlation degree and topological structure between nodes (e.g. gene, SNP, etc.) from biological network. Despite, Pathway Effect Measures (PEM) with a case-control design [13] fully utilizes the correlation relationship between nodes, it only considers the chain-specific effects and encounters difficulties in non-linear and interaction models. Specially, the estimation of chain-specific effect is different from the path-specific effect extracted from a complex network, the former one does not take into account the influence of other adjacent paths or nodes (e.g. parent or child nodes). Besides the chain effect is solely marginal statistical association, but the specific path effect is developed based on causal inference and needs to adjust for necessary covariates affecting specific path. Pearl [18] firstly defined path-specific effects in the terms of causal diagrams. And Avin et al. [19] provided general necessary and sufficient conditions for their identification for a single exposure and outcome, while Shpitser [20] generalized these definitions and conditions to settings with multiple exposures, multiple outcomes, and possible hidden variables. Miles [21] developed a suite of multiply-robust, semi-parametric efficient estimator for the path-specific effect. However, these methods tend to require a number of strict assumptions which are difficult to be verified in practical applications, especially for complex network structures in biological fields.

In order to reduce the computational burden, we proposed a series of simplification process for the topology structure of complex networks. Of note, the nodes on specific path are only influenced by their parent nodes according to Markov Independence property. After simplification, the path-specific effect statistic PSE is estimated under two conditions to detect the differential specific paths. Therefore, the statistic PSE combined the causal effect calculation under causal inference framework with the network comparison in systems epidemiology designs. To assess the performances of the statistic PSE, theoretical proofs and statistical simulations are conducted to evaluate the stability of type I error and power, and a real gene expression data in Mammalian Target Of Rapamycin (mTOR) pathway on survival time of glioblastoma multiforme (GBM) patients are further analyzed to validate the practicability of PSE statistic.

## Application

Gliomas are the most common type of primary brain tumor, and are histologically differentiated as astrocytomas, oligodendrogliomas, and ependymomas. The World Health Organization (WHO) classifies central nervous system tumors into four different grades: I, II, III and IV. Grade IV glioblastoma multiforme (GBM) is the most frequent, devastating, and malignant astrocytic glioma. It is characterized by a high degree of cellularity, vascular proliferation, tumor cell chemoresistance, and necrosis. Even after neurosurgical resection, followed by aggressive chemotherapy and radiotherapy, GBM is still considered an incurable malignancy. No effective treatment agent against GBM has been identified [22–24].

**Fig. 1:**
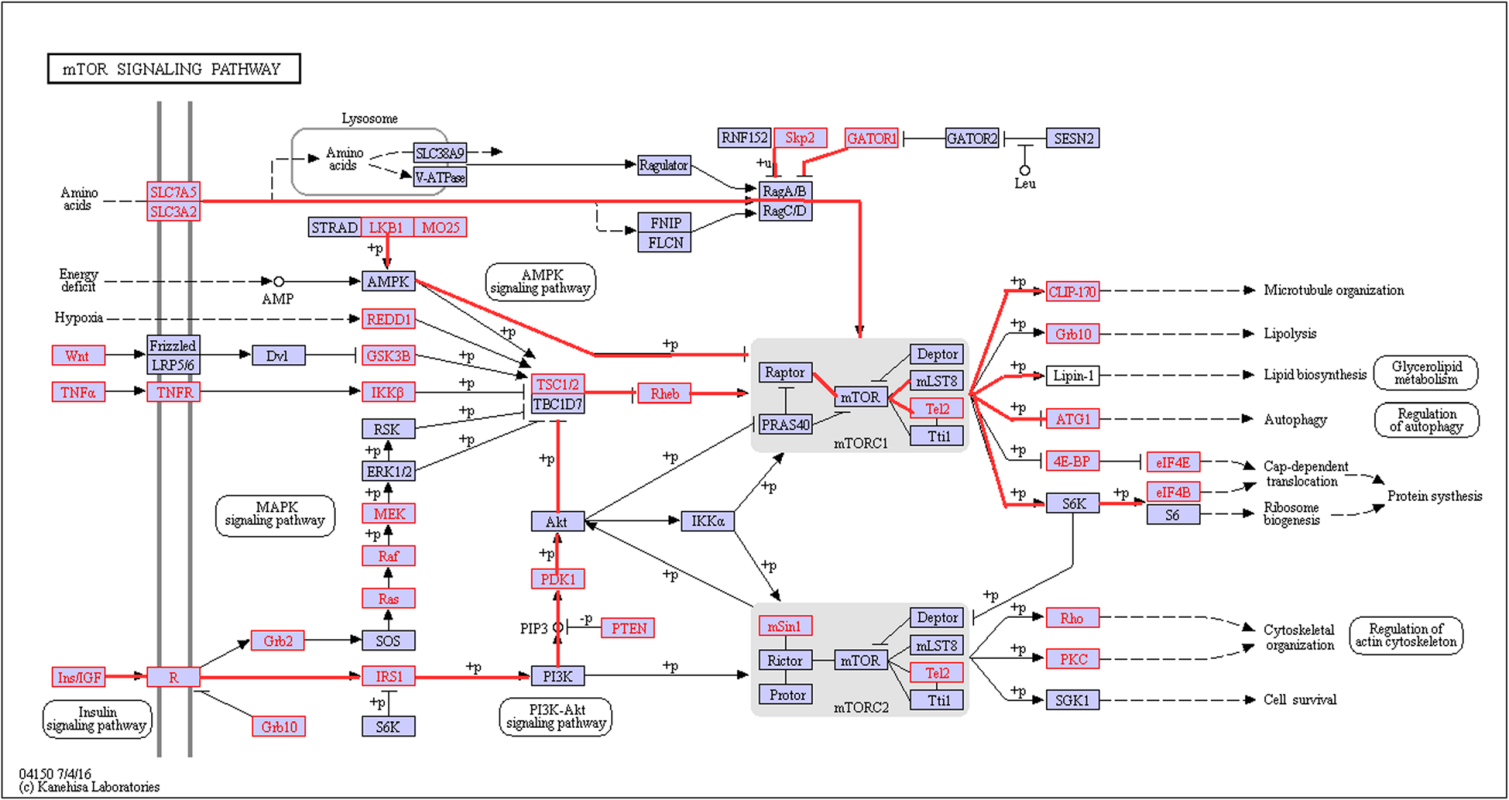
The mTOR signal pathway. Genes colored by red are available in TCGA dataset. The pathways with red line are the statistical significance

The proposed PSE statistic was applied to analyze gene expression data in Mammalian target of rapamycin (mTOR) signal pathway (Fig. 1) of 461 white people from TCGA datasets containing 12,071 genes by comparing the survival time (i.e. more VS. less than the mean survival time), and 39 genes are successfully mapped to this signaling pathway. The pathway mTOR, a key mediator of phosphatidyl-inositol-3-kinase (PI3K) signaling, has emerged as a compelling molecular target in glioblastoma patients, although clinical efforts to target mTOR have not been successful. Here, we support the evidence demonstrating that mTOR is a compelling molecular target for the survival event with GBM. It was approved by ethical committee of Medical Ethical Committee of Qilu Hospital, Shandong University, China.

## Results

### Simulation

#### Type I error rate

**Fig. 2:**
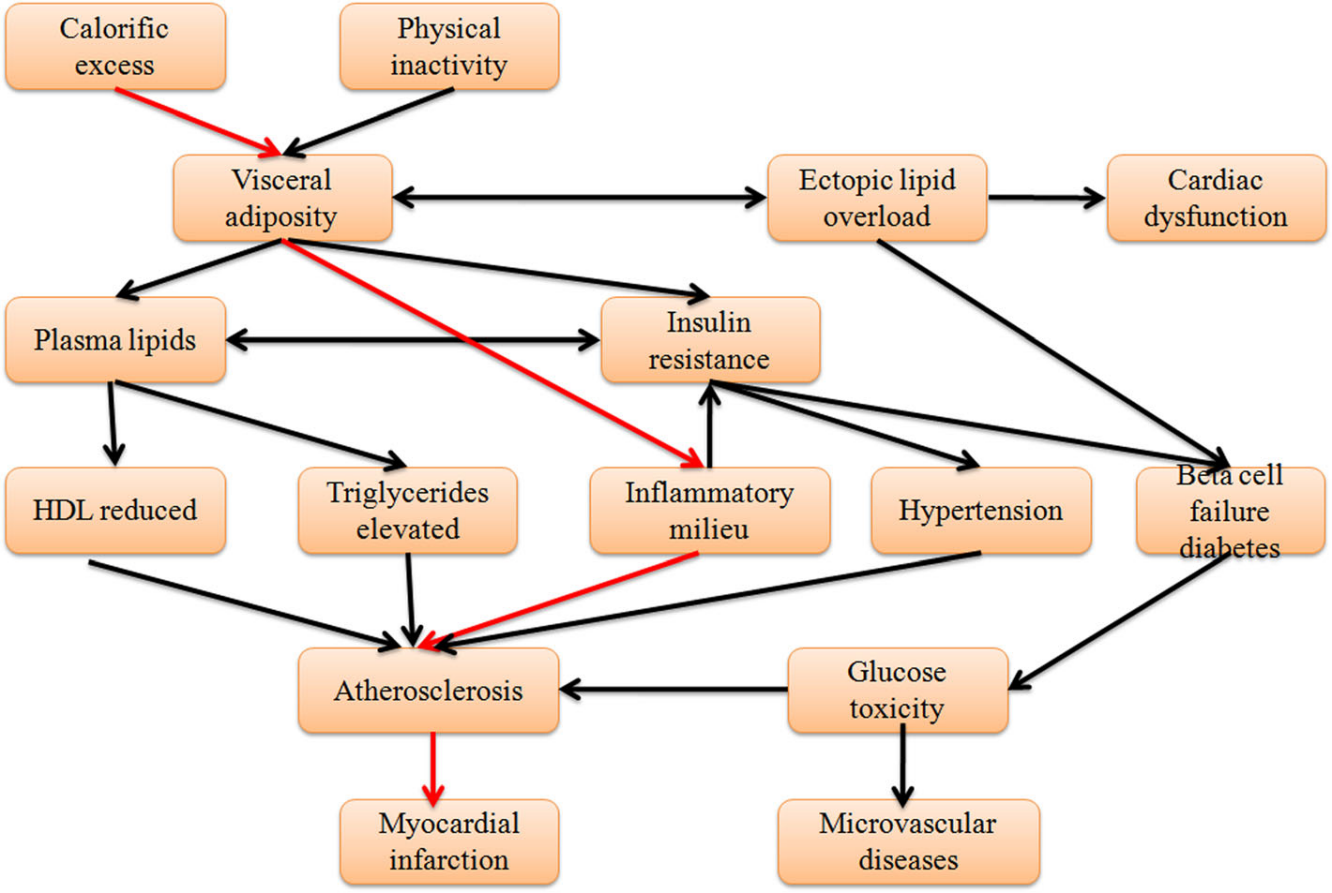
A complex biological network on Myocardial Infarction

Tables 1 and 2 showed the type I error rates of total causal effects (TCE) of Calorific Excess on Myocardial Infarction and the path-specific effects along selected the specific path: Calorific Excess→Visceral Adiposit→Inflammatory Milieu→Atherosclerosis→Myocardial Infarction (Fig. 2), respectively. Table 1 revealed that the type I error rates of five methods are close to the given nominal level (*α* = 0.05) when sample size reached 1000 for total causal effects. While Table 2 illustrated that only permutation tests remained stable at the nominal level of 0.05, other methods deviated from the 0.05 nominal level, when sample size reached 1000 for path-specific effects. Therefore, PSE statistic with permutation tests had better performances for testing total causal effect or path-specific effect.

**Table 1:** Type I error rates of five non-parameter methods varying across sample sizes for total causal effect

Sample	Permutation	Normal CI	Basic CI	Percentile CI	BS CI
200	0.060	0.070	0.065	0.095	0.115
400	0.080	0.070	0.065	0.075	0.085
600	0.035	0.050	0.055	0.060	0.070
800	0.055	0.050	0.055	0.050	0.070
1000	0.040	0.045	0.040	0.045	0.055

**Table 2:** Type I error rates of five non-parameter methods varying across sample sizes for path-specific effect

Sample	Permutation	Normal CI	Basic CI	Percentile CI	BS CI
200	0.005	0.000	0.000	0.000	0.000
400	0.035	0.000	0.000	0.000	0.005
600	0.045	0.000	0.000	0.000	0.010
800	0.070	0.000	0.000	0.000	0.010
1000	0.055	0.000	0.000	0.000	0.005

### Statistical power

Table 3 showed that the powers of five methods almost remained invariant for testing total causal effects when varying across the average causal effects of edges on specific path and given the fixed effect difference *δ* = 1 (Case group vs. Control group). Table 4 showed the power of permutation tests got larger for path-specific effects when the average causal effect of each edge on target path became larger.

**Table 3:** The powers of five methods varying across effects of each edge on target path for total causal effect

Difference *δ*	Effect sizes	Permutation	Normal CI	Basic CI	Percentile CI	BS CI
*δ* = 1	0.2 vs 1.2	0.075	0.080	0.075	0.100	0.100
*δ* = 1	0.4 vs 1.4	0.045	0.045	0.035	0.050	0.055
*δ* = 1	0.6 vs 1.6	0.060	0.065	0.070	0.070	0.065
*δ* = 1	0.8 vs 1.8	0.045	0.050	0.060	0.055	0.055
*δ* = 1	1.0 vs 2.0	0.035	0.040	0.045	0.045	0.045

**Table 4:** The power of PSE with permutation tests varying across effects of target path effect for path-specific effect

*δ*	*ca* → *vi*	*vi* → inf	inf → *at*	*at* → *my*	Power
*δ* = 1	0.2 vs 1.2	0.2 vs 1.2	0.2 vs 1.2	0.2 vs 1.2	0.790
*δ* = 1	0.4 vs 1.4	0.4 vs 1.4	0.4 vs 1.4	0.4 vs 1.4	0.920
*δ* = 1	0.6 vs 1.6	0.6 vs 1.6	0.6 vs 1.6	0.6 vs 1.6	0.960
*δ* = 1	0.8 vs 1.8	0.8 vs 1.8	0.8 vs 1.8	0.8 vs 1.8	0.990
*δ* = 1	1.0 vs 2.0	1.0 vs 2.0	1.0 vs 2.0	1.0 vs 2.0	1.000

Besides, Tables 5 and 6 showed that varying across the effect difference *δ* of path-specific effects in case and control group, the powers of total causal effect and path-specific effect obviously elevated. Furthermore, we performed sensitivity analysis to observe whether the PSE statistic would be influenced by the parent nodes or child nodes of nodes on specific path. Tables 7 and 8 revealed that in most cases path-specific effect statistic PSE was not influenced by effects of their parent nodes or child nodes. According to above simulation performances, our proposed PSE with permutation test had better performances and kept robust in sensitivity analysis.

**Table 5:** The powers of five methods varying across effect difference *δ* for total causal effect

*δ*	Effect sizes	Permutation	Normal CI	Basic CI	Percentile CI	BS CI
*δ* = 0.5	0.5 vs 1.0	0.045	0.045	0.050	0.055	0.060
*δ* = 1.0	0.5 vs 1.5	0.045	0.050	0.060	0.070	0.070
*δ* = 1.5	0.5 vs 2.0	0.055	0.060	0.050	0.055	0.065
*δ* = 2.0	0.5 vs 2.5	0.080	0.110	0.125	0.120	0.130
*δ* = 2.5	0.5 vs 3.0	0.350	0.380	0.380	0.385	0.365
*δ* = 3.0	0.5 vs 3.5	0.700	0.735	0.765	0.725	0.730

**Table 6:** The power of PSE with Permutation tests varying across the effect difference *δ* under two conditions for path-specific effect

*δ*	*ca* → *vi*	inf → *at*	*at* → *my*	Power of PSE
*δ* = 0.5	0.5 vs 1.0	0.5 vs 1.0	0.5 vs 1.0	0.395
*δ* = 1.0	0.5 vs 1.5	0.5 vs 1.5	0.5 vs 1.5	0.920
*δ* = 1.5	0.5 vs 2.0	0.5 vs 2.0	0.5 vs 2.0	0.970
*δ* = 2.0	0.5 vs 2.5	0.5 vs 2.5	0.5 vs 2.5	0.945
*δ* = 2.5	0.5 vs 3.0	0.5 vs 3.0	0.5 vs 3.0	0.955
*δ* = 3.0	0.5 vs 3.5	0.5 vs 3.5	0.5 vs 3.5	0.970

**Table 7:** The performances of PSE with permutation tests varying across the effects of edges from parent nodes not on target path to nodes on target path

Effect difference	*ph* → *vi*	hdl → *at*	*tr* → *at*	*hy* → *at*	*glu* → *at*	Power
*δ* = 1.0	0.2 vs 1.2	0.2 vs 1.2	0.2 vs 1.2	0.2 vs 1.2	0.2 vs 1.2	0.925
*δ* = 1.0	0.4 vs 1.4	0.4 vs 1.4	0.4 vs 1.4	0.4 vs 1.4	0.4 vs 1.4	0.965
*δ* = 1.0	0.6 vs 1.6	0.6 vs 1.6	0.6 vs 1.6	0.6 vs 1.6	0.6 vs 1.6	0.960
*δ* = 1.0	0.8 vs 1.8	0.8 vs 1.8	0.8 vs 1.8	0.8 vs 1.8	0.8 vs 1.8	0.935
*δ* = 1.0	1.0 vs 2.0	1.0 vs 2.0	1.0 vs 2.0	1.0 vs 2.0	1.0 vs 2.0	0.935

**Table 8:** The performances of PSE with permutation tests varying across the effect differences of edges from nodes on target path to their child nodes not on target path

Effect differences	*vi* → *pl*	v*i* → *ins*	inf → *ins*	Power
*δ* = 1.0	0.2 vs 1.2	0.2 vs 1.2	0.2 vs 1.2	0.923
*δ* = 1.0	0.4 vs 1.4	0.4 vs 1.4	0.4 vs 1.4	0.915
*δ* = 1.0	0.6 vs 1.6	0.6 vs 1.6	0.6 vs 1.6	0.960
*δ* = 1.0	0.8 vs 1.8	0.8 vs 1.8	0.8 vs 1.8	0.960
*δ* = 1.0	1.0 vs 2.0	1.0 vs 2.0	1.0 vs 2.0	0.965

**Fig. 3:**
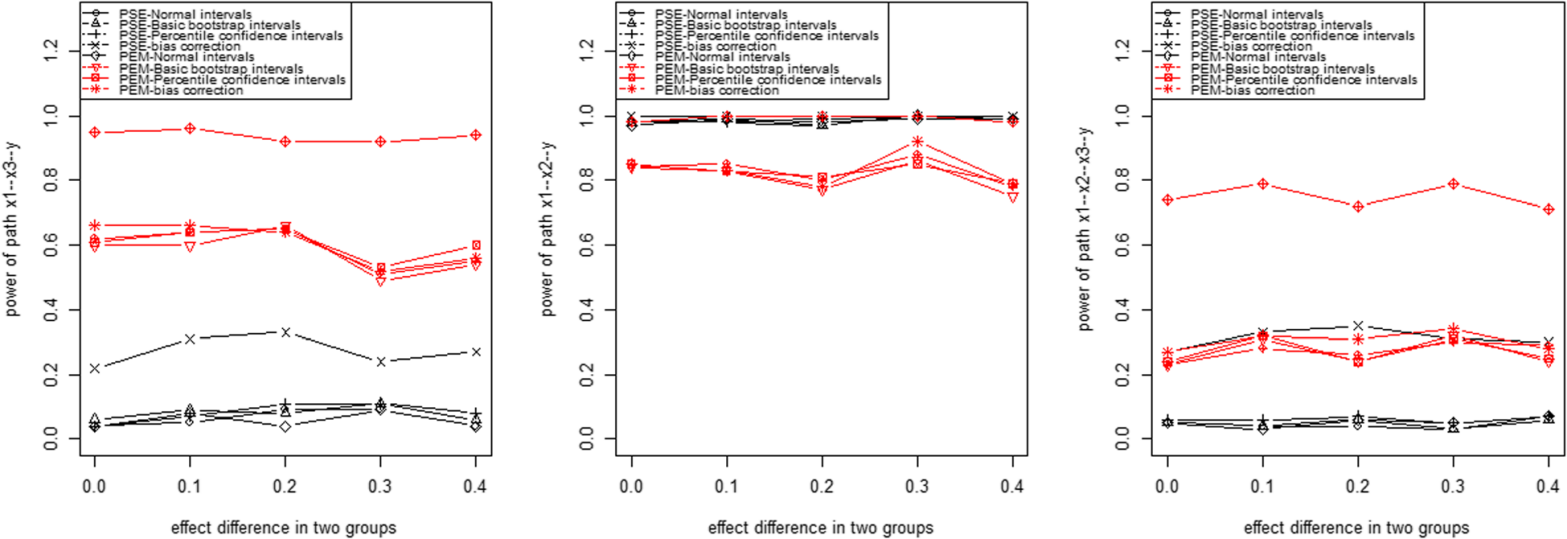
The performances of PSE and PEM statistics for detecting three pathways

For the scenario of continuous variables, when comparing with the PEM [13] statistic with Bootstrap tests, our proposed PSE statistics accurately detected the differential pathway effects X_1_ → X_2_ → Y linking X_1_ and Y under two conditions for the fixed effect difference. The PEM with Bootstrap tests detected some false positive specific pathways (Fig. 3).

### Application results

Mammalian Target Of Rapamycin (*mTO*R), a key mediator of phosphatidyl-inositol-3-kinase (*PI3K*) signaling, has emerged as a compelling molecular target in glioblastoma patients, although clinical efforts to target mTOR have not been successful [22–24]. Figure 1 showed the *mTOR* pathway from KEGG dataset (www.kegg.jp) that have been verified to be associated with the survival time of glioblastoma multiforme (GBM). The data (sample size *N* = 461 white people) of this pathway (Fig. 1) containing 39 genes in red boxes were downloaded from “The Cancer Genome Atlas” (TCGA, https://cancergenome.nih.gov/). We stratified the path-specific effects according to the survival time *T* (*T* = 1 if survival time larger than mean survival time 16.65 months of patients diagnosed with GBMs, otherwise *T* = 0) and adjusted for confounders including age and sex in white people.

Furthermore, we found 14 specific pathways with statisical significance (Table 9) contributing to GBM and corresponding 17 genes, *SLC7A5*, *mLST8*, *Lipin-*1, *Tel2*, *CLIP-170*, *ATG1*, *SLC3A2*, *RNF152*, *eIF4B*, *GATOR1*, *STRAD*, *IGFR*, *IRS*1, *PDK1*, *TSC1/6*, *Rheb*. These genes have also been verified in many studies. The pathway *mTOR* works through the *PI3K* pathway through 2 important complexes, each characterized by distinct binding partners that confer different activities. In complex with *PRAS40*, raptor, and *mLST8/GbL*, *mTOR* works as a downstream activator of *PI3K/Akt* signaling, associating growth factor signals with protein translation, cell growth, proliferation, and survival state. This complex is known as *mTORC1*. In complex with rictor, *mSIN1*, *protor*, and *mLST8* (*mTORC2*), *mTOR* works as an upstream effector of *Akt* [24]. Upon growth factor receptor-mediated activation of PI3K, *Akt* is recruited to the membrane through the interaction of its pleckstrin homology domain with *PIP3*, thus exposing its activation loop and enabling phosphorylation at threonine 308 (*Thr308*) via the constitutively active phosphoinositide dependent protein kinase 1 (*PDK1*) [25–27]. Akt activates *mTORC*1 via inhibitory phosphorylation of *TSC2*, which along with *TSC1*, negatively regulates *mTORC1* through inhibiting the *Rheb GTPase*, a positive regulator of *mTORC*1. *mTORC1* impairs *PI3K* activation in growth factor-stimulated cells by downregulating *IRS 1* and 2 and *PDGFR* [24, 28, 29]. The pathway *mTORC1* regulates *SREBP* via regulating the nuclear entry of lipin 1, a phosphatidic acid phosphatase. Dephosphorylated, nuclear, catalytically active lipin 1 help nuclear remodeling and mediate the effects of *mTORC1* on *SREBP* target gene, *SREBP* promoter activity, and nuclear *SREBP* protein abundance. Inhibition of *mTORC1* in the liver significantly impairs *SREBP* function and makes mice resistant, in a lipin 1-dependent fashion, to the hepatic steatosis and hypercholesterolemia induced by a high fat and cholesterol diet. These findings developed lipin 1 as a key component of the *mTORC1-SREBP* pathway [25]. Some studies provided evidence that *ATG1* was the preferred translation initiation site in *8MGBA*, and that endogenous *SETMAR* were very stable proteins [25]. In summary these data allowed us to propose that endogenous *SETMAR* proteins can contain the α-peptide in their N-terminal part, at least at some stages of *GBM* biogenesis [26]. The gene *Rheb* acts downstream of *TSC1/TSC2* and upstream of *mTOR* to regulate cell growth. Both *IGF-IR* and *IGF-IIR* were overexpressed in *GBMs* compared with normal brain (*P* < 10(− 4) and *P* = 0.002, respectively). Moreover, with regard to standard clinical factors, *IGF-IR* positivity was identified as an independent prognostic factor associated with shorter survival (*P* = 0.016) and was associated with a less favourable response to temozolomide [27]. The pathway *mTOR* regulates *eIF4B* phosphorylation in response to amino-acid refeeding [30]. Glioblastoma is the most aggressive brain cancer with the poor survival rate. A microRNA, *miR-451*, and its downstream molecules, *STRAD*, are known to play a centrol role in regulating a biochemical balance between rapid proliferation and invasion in the presence of metabolic stress in microenvironment [31].

**Table 9:** The detected pathways with statistical significance contributing to survival time in GBM patients

*Path list*	*PSE*	*SE*	*P* value
SLC7A5 → mLST8 → Lipin-1	2.11046	0.995	0.017
SLC7A5 → Tel2 → CLIP-170	1.977606	0.99	0.023
SLC7A5 → Tel2 → Lipin-1	1.718378	0.8765	0.025
SLC7A5 → Tel2 → ATG1	2.595217	1.077	0.008
SLC3A2 → mLST8 → Lipin-1	3.461764	1.203	0.002
SLC3A2 → Tel2 → CLIP-170	2.021598	0.93	0.015
SLC3A2 → Tel2 → Lipin-1	1.94616	0.966	0.022
SLC3A2 → Tel2 → ATG1	2.742903	1.198	0.011
RNF152 → mLST8 → eIF4B	−2.32003	1.214	0.028
GATOR1 → Tel2 → CLIP-170	1.806073	1.01	0.037
GATOR1 → Tel2 → ATG1	1.791135	1.02	0.04
STRAD→Tel2 → CLIP-170	1.754709	1.029	0.044
IGF → R → IRS1 → PDK1 → TSC1/6 → Rheb→mLST8 → eIF4B	1.143228	0.691	0.049
IGF → IRS1 → PDK1 → mLST8 → eIF4B	1.151496	0.675	0.044

## Discussion

System epidemiology couples traditional epidemiology with modern high-throughput technologies which seek to integrate pathway-based (or network-based) analysis into observational study designs to enhance the understanding of biological processes in the human organism. It provides a ways to organize and study the inter-dependencies of factors (e.g., genes, proteins, metabolites) at a human population level. Within this framework, the identification of pathways effects responsible for specific diseases has been one of the essential tasks. In the framework of bioinformatics, various methods existed for inferring biological networks aiming to mine underlying networks for identifying biological modules, clustering interactions, and topological features of the network such as degree and betweenness centrality [32–34]. Despite these procedures for distinguishing specific pathway (or network) topology between different disease status, statistical inference at a population level remains unsolved, and further development is still necessary.

**Fig. 4:**
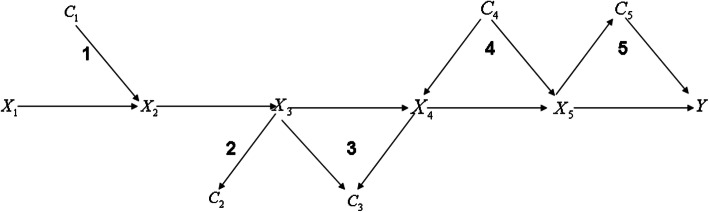
Simplified complex network. 1) single conflux path; 2) single diffluent path; 3) colliding path by two diffluent paths; 4) confounding path by two conflux path; 5) mediator path linking by a diffluent path and conflux path

Because, in practice, complexity of network tend to render it difficult to accurately detect the pathway contribution to disease, the simplification process of complex network is very crucial for identifying the target pathways. Based on the aim of identification of path-specific effects, we proposed a series of simplification process to simplify and abstract the topology structure of complex network (Fig. 4). Of note, the nodes of path-specific is only influenced by their parent nodes according to Markov Independence property. This simplification process greatly reduce the complexity of network structure and maintain the key factors affecting the target specific paths. Currently in the field of causal inference, most methods mainly focus on the simple and easily understandable causal diagrams, but the simplification is the crucial first step to feasibly serve to real world.

After simplification, calculation and tests of path-specific effect also became feasible. We proposed a non-parameter riverway conflux-based non-parameter causal diagram model for identifying the path-specific effects in systems epidemiology. Simulation studies revealed our proposed PSE with permutation tests could efficiently identify the statistically different pathways. Table 1 revealed that the type I error of five methods are close to the given nominal level (*α* = 0.05) when sample Jsize reached 1000. While Table 2 illustrated that only PSE with permutation tests remained stable, other methods deviated from the nominal level 0.05, when sample size were larger than 1000. Therefore, PSE statistic with permutation tests had better performances for testing total causal effect or path-specific effect. Table 3 revealed that the powers of five methods almost remained invariant for total causal effect when the average causal effects *β* of edge on the specific path became larger and the effect difference *δ* was set to 1. On the contrary, the power of permutation tests got larger and was close to 1 for the path-specific effect as average causal effect went up. Besides, Tables 5 and 6 revealed that varying across the effect differences *δ*, the power on total causal effect and path-specific effect obviously elevated. Furthermore, we performed sensitivity analysis to observe that in most situations, PSE statistic would be not influenced by the parent nodes or child nodes of nodes on specific path (Tables 7 and 8).

In application analysis, the proposed typical PSE statistic was applied to analyze gene expression data in Mammalian target of rapamycin (mTOR) signal pathway (Fig. 1) of 461 white people from TCGA datasets containing 12,071 genes, 39 genes are successfully mapped to this signaling pathway. The pathway mTOR, a key mediator of phosphatidyl-inositol-3-kinase (*PI3K*) signaling, has emerged as a compelling molecular target in glioblastoma patients, although clinical efforts to target mTOR have not been successful. Here, we support the evidence demonstrating that mTOR is a compelling molecular target in GBM.

Figure 1 showed the mTOR pathway from KEGG dataset (www.kegg.jp) that have been verified to be associated with the survival time of glioblastoma multiforme (GBM) [32–34]. The data (sample size *N* = 461 white people) concerning this pathway containing 39 genes in red boxes were downloaded from “The Cancer Genome Atlas” (TCGA). Our studies results obtained 14 statistically significant positive pathways (Table 9). We stratified the path-specific effects according to the survival time *T* (*T* = 1 if survival time larger than mean survival time 16.65 months of patients diagnosed with GBMs, otherwise *T* = 0) and adjusted for confounders including age and sex. Furthermore, we found 14 statistically positive specific pathways (Table 9) and most gene have been verified in many studies.

## Conclusion

In the framework of systems epidemiology, the proposed permutation-based PSE are valid and powerful for detecting the specific pathway effect contributing to disease, thus potentially providing new insights and ways to unlock the black box of disease mechanism.

## Methods

### Complex network simplification rules (Fig. 4)

For specific path in complex network, we proposed some rules to simplify complex networks and extract specific path from complex network. Remove irrelevant variables from the causal diagram including (i) no causal effect on the variables of target path (e.g. C_1_ in Fig. 4) and (ii) no causal effect on any variable in the adjustment set (e.g. C_2_ in Fig. 4). These variables will not induce spurious association so can be ignored. Besides considering the influence of direct and indirect causal effect, we need to merge all direct and indirect causal paths between two variables. Finally, confounding paths or non-causal path remained in simplified causal diagram paths.

### Path-specific effect statistic PSE

**Fig. 5:**
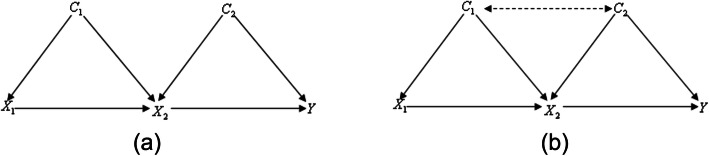
Causal diagrams for specific path *X*_1_ → *X*_2_ → *Y* with *C* = (*C*_1_, *C*_2_). **a***C*_1_ is independent of *C*_2_; **b***C*_1_ is associated with *C*_2_

For the sake of illustration, we take the specific path *X*_1_ → *X*_2_ → *Y* (Fig. 5) as an example. We wish to calculate the path-specific effect based on the average causal effect. Firstly, according to the expectation dependence of *X*_1_ and *Y*, we have

1 $$ E\left(Y\left|{x}_1^{\prime}\right.\right)-E\left(Y\left|{x}_1^{{\prime\prime}}\right.\right)=-{\int}_{-\infty}^{+\infty}\left\{F\left(y\left|{x}_1^{\prime}\right.\right)-F\left(y\left|{x}_1^{{\prime\prime}}\right.\right)\right\} dy $$

and $$ \frac{\partial E\left(Y|{x}_1\right)}{\partial {x}_1}=-{\int}_{-\infty}^{+\infty}\frac{\partial F\left(y|{x}_1\right)}{\partial {x}_1} dy $$.

Take the causal diagram depicted in Fig. 5a as a special case, the average causal effect (ACE) of *X*_1_ on *Y* is used to compare the effects of two different levels of *X*_1_, i.e., ${x}_1^{\prime }$ and ${x}_1^{{\prime\prime} }$. Since *p*(*c*| *do*(*x*_1_)) = *p*(*c*) and *p*(*y*| *c*, *do*(*x*_1_)) = *p*(*y*| *c*, *x*_1_), we obtain

$$ ACE\left({X}_1\to Y| do\left({x}_1^{\prime}\right), do\left({x}_1^{{\prime\prime}}\right)\right)=\int p(c)E\left(Y|c,{x}_1^{\prime}\right) dc-\int p(c)E\left(Y|c,{x}_1^{{\prime\prime}}\right) dc. $$

Similarly, we obtain the ACE of *X*_1_ on *X*_2_ as

$$ ACE\left({X}_1\to {X}_2| do\left({x}_1^{\prime}\right), do\left({x}_1^{{\prime\prime}}\right)\right)=\int p(c)E\left({X}_2|c,{x}_1^{\prime}\right) dc-\int p(c)E\left({X}_2|c,{x}_1^{{\prime\prime}}\right) dc. $$

From *p*(*c*| *do*(*x*_2_)) = *p*(*c*) and *p*(*y*| *c*, *do*(*x*_2_)) = *p*(*y*| *c*, *x*_2_), we have

$$ ACE\left\{{X}_2\to Y| do\left({x}_2^{\prime}\right), do\left({x}_2^{{\prime\prime}}\right)\right\}=\int p(c)\left\{E\left(Y|c,{x}_2^{\prime}\right)-E\left(Y|c,{x}_2^{{\prime\prime}}\right)\right\} dc $$

### Case of continuous variables

We first consider the case of continuous variables depicted in Fig. 5b. By *C* = (*C*_1_,  *C*_2_), *X*_1_ ⊥ *Y* ∣ (*C*_2_, *X*_2_), we obtain

$$ E\left(Y|c,{x}_1\right)=\int E\left(Y|c,{x}_1,{x}_2\right)p\left({x}_2|c,{x}_1\right){dx}_2=\int E\left(\mathrm{Y}|c,{x}_2\right)p\left({x}_2|c,{x}_1\right){dx}_2=\int E\left(Y|c,{x}_2\right)\frac{\partial F\left({x}_2|c,{x}_1\right)}{\partial {x}_2}{dx}_2 $$

Applying integration by parts and definite integration:

2 $$ {\displaystyle \begin{array}{c}E\left(Y|c,{x}_1^{\prime}\right)-E\left(Y|c,{x}_1^{\prime \prime}\right)=\int \frac{\partial \left\{F\left({x}_2|c,{x}_1^{\prime}\right)-F\left({x}_2|c,{x}_1^{\prime \prime}\right)\right\}}{\partial {x}_2}E\left(Y|c,{x}_2\right){dx}_2\\ {}=-\int \left\{F\left({x}_2|c,{x}_1^{\prime}\right)-F\left({x}_2|c,{x}_1^{\prime \prime}\right)\right\}\frac{\partial E\left(Y|c,{x}_2\right)}{\partial {x}_2}{dx}_2\\ {}=-\int \left\{F\left({x}_2|{c}_1,{c}_2,{x}_1^{\prime}\right)-F\left({x}_2|{c}_1,{c}_2,{x}_1^{\prime \prime}\right)\right\}\frac{\partial E\left(Y|{c}_1,{c}_2,{x}_2\right)}{\partial {x}_2}{dx}_2\\ {}=-\int \left\{F\left({x}_2|{c}_1,{c}_2,{x}_1^{\prime}\right)-F\left({x}_2|{c}_1,{c}_2,{x}_1^{\prime \prime}\right)\right\}\frac{\partial E\left(Y|{c}_2,{x}_2\right)}{\partial {x}_2}{dx}_2\end{array}} $$

If the distribution dependence is non-decreasing, so is the expectation dependence.

**Theorem 1**: For the specific path *X*_1_ → *X*_2_ → *Y* with confounders *C*, any ${x}_2^{\prime }>{x}_2^{{\prime\prime} }$, we have

$$ ACE\left\{{X}_1\to \mathrm{Y}| do\left({x}_1^{\prime}\right), do\left({x}_1^{{\prime\prime}}\right)\right\}=\frac{ACE\left\{{X}_1\to {X}_2| do\left({x}_1^{\prime}\right), do\left({x}_1^{{\prime\prime}}\right)\right\} ACE\left\{{X}_2\to Y| do\left({x}_2^{\prime}\right), do\left({x}_2^{{\prime\prime}}\right)\right\}}{x_2^{\prime }-{x}_2^{{\prime\prime} }} $$

if satisfy the conditions:

1. $$ \frac{\partial E\left(Y|c,{x}_2\right)}{\partial {x}_2}\perp C $$
  or
2. $$ \left[E\left({X}_2|c,{x}_1^{\prime}\right)-E\left({X}_2|c,{x}_1^{{\prime\prime}}\right)\right]\perp C $$

Proof: For condition 1, according to Eqs. 1 and 2, we have

$$ {\displaystyle \begin{array}{c} ACE\left\{{X}_1\to Y| do\left({x}_1^{\prime}\right), do\left({x}_1^{{\prime\prime}}\right)\right\}=\int p(c)\int \left\{F\left({x}_2|c,{x}_1^{{\prime\prime}}\right)-F\left({x}_2|c,{x}_1^{\prime}\right)\right\}\frac{\partial E\left(Y|c,{x}_2\right)}{\partial {x}_2}{dx}_2 dc\\ {}=\int p(c)\frac{\partial E\left(Y|c,{x}_2\right)}{\partial {x}_2}\int \left\{F\left({x}_2|c,{x}_1^{{\prime\prime}}\right)-F\left({x}_2|c,{x}_1^{\prime}\right)\right\}{dx}_2 dc\\ {}=\int p(c)\frac{\partial E\left(Y|c,{x}_2\right)}{\partial {x}_2}\int \left\{F\left({x}_2|c,{x}_1^{{\prime\prime}}\right)-F\left({x}_2|c,{x}_1^{\prime}\right)\right\}{dx}_2 dc\\ {}=\int p(c)\frac{\partial E\left(Y|c,{x}_2\right)}{\partial {x}_2}\left\{E\left\{{X}_2|c,{x}_1^{\prime}\right\}-E\left({X}_2|c,{x}_1^{{\prime\prime}}\right)\right\} dc\\ {}=\frac{\partial E\left(Y|c,{x}_2\right)}{\partial {x}_2} ACE\left({X}_1\to {X}_2| do\left({x}_1^{\prime}\right), do\left({x}_1^{{\prime\prime}}\right)\right)\end{array}} $$

By Eq. 2, the effect of *X*_2_ on *Y* can be written as

$$ {\displaystyle \begin{array}{c} ACE\left\{{X}_2\to Y| do\left({x}_2^{\prime}\right), do\left({x}_2^{{\prime\prime}}\right)\right\}=\int p(c)E\left({X}_2|c,{x}_2^{\prime}\right)-E\left({X}_2|c,{x}_2^{{\prime\prime}}\right) dc\\ {}=\int p(c)\frac{\partial E\left(Y|c,{x}_2\right)}{\partial {x}_2}\left({x}_2^{\prime }-{x}_2^{{\prime\prime}}\right) dc\\ {}=\frac{\partial E\left(Y|c,{x}_2\right)}{\partial {x}_2}\left({x}_2^{\prime }-{x}_2^{{\prime\prime}}\right)\end{array}} $$

From above two equations, we obtain

$$ ACE\left\{{X}_1\to \mathrm{Y}| do\left({x}_1^{\prime}\right), do\left({x}_1^{{\prime\prime}}\right)\right\}=\frac{ACE\left\{{X}_1\to {X}_2| do\left({x}_1^{\prime}\right), do\left({x}_1^{{\prime\prime}}\right)\right\} ACE\left\{{X}_2\to Y| do\left({x}_2^{\prime}\right), do\left({x}_2^{{\prime\prime}}\right)\right\}}{x_2^{\prime }-{x}_2^{{\prime\prime} }} $$

Similarly, for condition 2, we can obtain

$$ {\displaystyle \begin{array}{c} ACE\left\{{X}_1\to Y| do\left({x}_1^{\prime}\right), do\left({x}_1^{{\prime\prime}}\right)\right\}=\int p(c)\int \left[F\left({x}_2|c,{x}_1^{{\prime\prime}}\right)-F\left({x}_2|c,{x}_1^{\prime}\right)\right]\frac{\partial E\left(Y|c,{x}_2\right)}{\partial {x}_2}{dx}_2 dc\\ {}=\int p(c)\frac{\partial E\left(Y|c,{x}_2\right)}{\partial {x}_2}\int \left[F\left({x}_2|c,{x}_1^{{\prime\prime}}\right)-F\left({x}_2|c,{x}_1^{\prime}\right)\right]{dx}_2 dc\\ {}=\int p(c)\frac{\partial E\left(Y|c,{x}_2\right)}{\partial {x}_2}\int \left[F\left({x}_2|c,{x}_1^{{\prime\prime}}\right)-F\left({x}_2|c,{x}_1^{\prime}\right)\right]{dx}_2 dc\\ {}=\int p(c)\frac{\partial E\left(Y|c,{x}_2\right)}{\partial {x}_2}\left[E\left\{{X}_2|c,{x}_1^{\prime}\right\}-E\left({X}_2|c,{x}_1^{{\prime\prime}}\right)\right] dc\\ {}=\left[E\left\{{X}_2|c,{x}_1^{\prime}\right\}-E\left({X}_2|c,{x}_1^{{\prime\prime}}\right)\right]{E}_C\left[\frac{\partial E\left(Y|c,{x}_2\right)}{\partial {x}_2}\right]\end{array}} $$

We also have

$$ {\displaystyle \begin{array}{c} ACE\left\{{X}_2\to Y| do\left({x}_2^{\prime}\right), do\left({x}_2^{{\prime\prime}}\right)\right\}=\int p(c)E\left(Y|c,{x}_2^{\prime}\right)-E\left(Y|c,{x}_2^{{\prime\prime}}\right) dc\\ {}=\left({x}_2^{\prime }-{x}_2^{{\prime\prime}}\right){E}_C\left[\frac{\partial E\left(Y|c,{x}_2\right)}{\partial {x}_2}\right]\end{array}} $$

and

$$ {\displaystyle \begin{array}{c} ACE\left\{{X}_1\to {X}_2| do\left({x}_1^{\prime}\right), do\left({x}_1^{{\prime\prime}}\right)\right\}=\int p(c)E\left({X}_2|c,{x}_1^{\prime}\right)-E\left({X}_2|c,{x}_1^{{\prime\prime}}\right) dc\\ {}=E\left({X}_2|c,{x}_1^{\prime}\right)-E\left({X}_2|c,{x}_1^{{\prime\prime}}\right)\end{array}} $$

Thus we obtain

3 $$ ACE\left\{{X}_1\to Y| do\left({x}_1^{\prime}\right), do\left({x}_1^{\prime \prime}\right)\right\}=\frac{ACE\left\{{X}_1\to {X}_2| do\left({x}_1^{\prime}\right), do\left({x}_1^{\prime \prime}\right)\right\} ACE\left\{{X}_2\to Y| do\left({x}_2^{\prime}\right), do\left({x}_2^{\prime \prime}\right)\right\}}{x_2^{\prime }-{x}_2^{\prime \prime }} $$

**Fig. 6:**
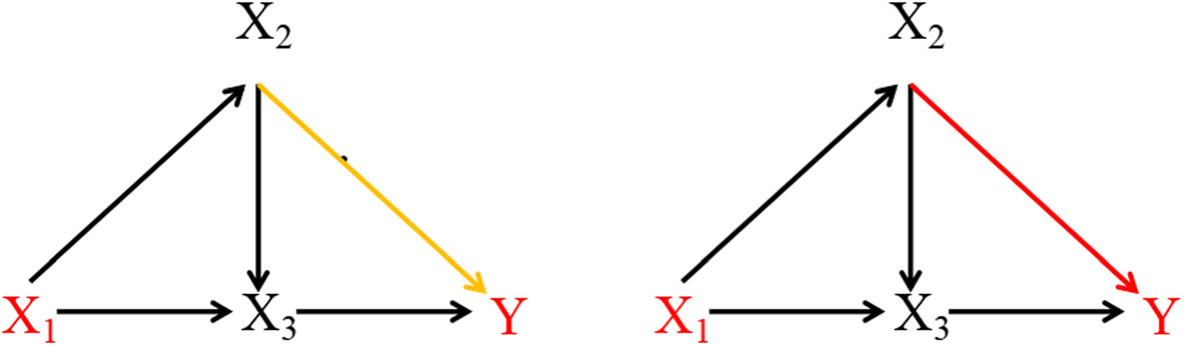
The causal graph linking X_1_ and Y in case and control groups. The dash colored line denotes the differential directed edge and X_1_ → X_2_ → Y is the unique differential path linking X_1_ and Y

In observational studies, in order to estimate the causal effect, we need to adjust for the parent nodes of nodes on the specific path. For instance, for the causal diagram in Fig. 6, according to the back-door criteria and do-calculus [18], the specific path effect of *X*_1_ → *X*_2_ → *Y*, we need to separately adjust for *C*_1_ and *C*_2_ by linear regression as follows,

4 $$ ACE\left\{{X}_1\to Y| do\left({x}_1^{\prime}\right), do\left({x}_1^{\prime \prime}\right)\right\}=\frac{\partial E\left({X}_2|{X}_1,{C}_1\right)}{X_1}\frac{\partial E\left(Y|{X}_2,{C}_2\right)}{X_2} $$

### Case of discrete variables

Similarly the results for case of discrete variables can be proved by substituding the partial differentiation and the integration into differencing between adjacent level and summation, respectively. We have

$$ {\displaystyle \begin{array}{c}E\left(Y|c,{x}_1\right)=\sum \limits_{m=0}^Mp\left({X}_2=m|c,{x}_1\right)E\left(Y|c,{X}_2=m\right)\\ {}=\sum \limits_{m=0}^M\left\{p\left({X}_2\le m|c,{x}_1\right)-p\left({X}_2\le m-1|c,{x}_1\right)\right\}E\left(Y|c,{X}_2=m\right)\\ {}=E\left(Y|c,{X}_2=M\right)-\sum \limits_{m=0}^{M-1}\left\{p\left({X}_2\le m|c,{x}_1\right)\right\}\left\{E\left(Y|c,{X}_2=m+1\right)-E\left(Y|c,{X}_2=m\right)\right\}\end{array}} $$

Thus, similar to Eq. 4, we obtain

$$ {\displaystyle \begin{array}{c} ACE\left\{{X}_1\to Y| do\left({x}_1^{\prime}\right), do\left({x}_1^{\prime \prime}\right)\right\}=\sum \limits_cp(c)E\left(Y|c,{x}_1^{\prime}\right)-\sum \limits_cp(c)E\left(Y|c,{x}_1^{\prime \prime}\right)\\ {}=\sum \limits_cp(c)\left\{\sum \limits_{m=0}^{M-1}P\left({X}_2\le m|c,{x}_1^{\prime}\right)-P\left({X}_2\le m|c,{x}_1^{\prime \prime}\right)\right\}\cdot \left\{E\left(Y|c,{X}_2=m+1\right)-E\left(Y|c,{X}_2=m\right)\right\}.\end{array}} $$

Similar to Eq. (1), we have

$$ ACE\left\{{X}_1\to {X}_2| do\left({x}_1^{\prime}\right), do\left({x}_1^{{\prime\prime}}\right)\right\}=\sum \limits_cp(c)\sum \limits_{x_2}\left\{p\left({X}_2\le {x}_2|c,{x}_1^{\prime}\right)-p\left({X}_2\le {x}_2|c,{x}_1^{{\prime\prime}}\right)\right\}. $$

From Eq. (2), for binary *X*_1_, *X*_2_ and *Y*, and *C* is a discrete variable which may have multiple values under the condition of [*E*(*Y*| *c*, *X*_2_ = *m* + 1) − *E*(*Y*| *c*, *X*_2_ = *m*)] ⊥ *C*. We have

$$ ACE\left\{{X}_1\to Y| do\left({x}_1^{\prime}\right), do\left({x}_1^{\prime \prime}\right)\right\}= ACE\left\{{X}_1\to {X}_2| do\left({x}_1^{\prime}\right), do\left({x}_1^{\prime \prime}\right)\right\} ACE\left\{{X}_2\to Y| do\left({x}_2^{\prime}\right), do\left({x}_2^{\prime \prime}\right)\right\}. $$

### Extension to the case of multiple mediators

In specific path *X*_1_ → *X*_2_ → ⋯ → *X*_*K*_ → *Y* with continuous confounders *C*, for any ${x}_i^{\prime }>{x}_i^{{\prime\prime} },\kern0.5em i=1,2,\cdots, K$, we have

$$ ACE\left\{{X}_1\to \mathrm{Y}| do\left({x}_1^{\prime}\right), do\left({x}_1^{\prime \prime}\right)\right\}=\frac{ACE\left\{{X}_1\to {X}_2| do\left({x}_1^{\prime}\right), do\left({x}_1^{\prime \prime}\right)\right\} ACE\left\{{X}_2\to {X}_3| do\left({x}_2^{\prime}\right), do\left({x}_2^{\prime \prime}\right)\right\}\cdots ACE\left\{{X}_K\to Y| do\left({x}_K^{\prime}\right), do\left({x}_K^{\prime \prime}\right)\right\}}{\prod \limits_{i=2}^K{x}_i^{\prime }-{x}_i^{\prime \prime }}. $$

For a discrete variable *C*, we have

$$ ACE\left\{{X}_1\to Y| do\left({x}_1^{\prime}\right), do\left({x}_1^{{\prime\prime}}\right)\Big)\right\}= ACE\left\{{X}_1\to {X}_2|c, do\left({x}_1^{\prime}\right), do\left({x}_1^{{\prime\prime}}\right)\right\} ACE\left\{{X}_2\to {X}_3|c, do\left({x}_2^{\prime}\right), do\left({x}_2^{{\prime\prime}}\right)\right\}\cdots ACE\left\{{X}_K\to Y|c, do\left({x}_K^{\prime}\right), do\left({x}_K^{{\prime\prime}}\right)\right\}. $$

In observational studies, according to back-door criteria and do-calculus [18], for the causal diagram in Fig. 5, the specific path effect of *X*_1_ → *X*_2_ → *Y* via adjusting for the binary parent nodes *C*_1_ and *C*_2_ is

$$ {\displaystyle \begin{array}{l} ACE\left\{{X}_1\to Y| do\left({X}_1={x}_1^{\prime}\right), do\left({X}_1={x}_1^{{\prime\prime}}\right)\right\}=\left[\sum \limits_{C_1}\left(P\left({X}_2|{X}_1={x}_1^{\prime },{C}_1\right)-P\Big({X}_2|{X}_1={x}_1^{{\prime\prime} },{C}_1\Big)\right)P\left({C}_1\right)\right]\\ {}\times \left[\sum \limits_{C_2}\left(P\left(Y|{X}_2={x}_2^{\prime },{C}_2\right)-P\Big(Y|{X}_2={x}_2^{{\prime\prime} },{C}_2\Big)\right)P\left({C}_2\right)\right]\end{array}}. $$

### Path-specific statistic for two comparisons

The proposed path-specific effect statistic (PSE) was based on product of average causal effect (ACE) of each directed edge, and took difference under two conditions (e.g., exposure vs. non-exposure, case vs. control). In order to identify the specific path *X*_1_ → *Y* the standardized path-specific effect in the exposure or case group was defined as

$$ {PSE}_1=\frac{ACE^1\left\{{X}_1\to Y| do\left({x}_1^{\prime}\right), do\left({x}_1^{{\prime\prime}}\right)\right\}}{S_{ACE^1\left\{{X}_1\to Y| do\left({x}_1^{\prime}\right), do\left({x}_1^{{\prime\prime}}\right)\right\}}}. $$

While for non-exposure or control group, the standardized path-specific effect was

$$ {PSE}_0=\frac{ACE^0\left\{{X}_1\to Y| do\left({x}_1^{\prime}\right), do\left({x}_1^{{\prime\prime}}\right)\right\}}{S_{ACE^0\left\{{X}_1\to Y| do\left({x}_1^{\prime}\right), do\left({x}_1^{{\prime\prime}}\right)\right\}}} $$

where ${ACE}^1\left\{{X}_1\to Y| do\left({x}_1^{\prime}\right), do\left({x}_1^{{\prime\prime}}\right)\right\}$, ${ACE}^0\left\{{X}_1\to Y| do\left({x}_1^{\prime}\right), do\left({x}_1^{{\prime\prime}}\right)\right\}$ denoted separately the average causal effect in case and control group, ${S}_{ACE^1\left\{{X}_1\to Y| do\left({x}_1^{\prime}\right), do\left({x}_1^{{\prime\prime}}\right)\right\}}$ and ${S}_{ACE^0\left\{{X}_1\to Y| do\left({x}_1^{\prime}\right), do\left({x}_1^{{\prime\prime}}\right)\right\}}$ denoted the standard error of ${ACE}^1\left\{{X}_1\to Y| do\left({x}_1^{\prime}\right), do\left({x}_1^{{\prime\prime}}\right)\right\}$ and ${ACE}^0\left\{{X}_1\to Y| do\left({x}_1^{\prime}\right), do\left({x}_1^{{\prime\prime}}\right)\right\}$ in case and control group, respectively. Hypothesis tests were performed to test whether the two path-specific effects had significant statistical difference. The null hypothesis and alternative hypothesis were separately equal to

$$ {H}_0:{PSE}_1={PSE}_0\kern0.5em {H}_1:{PSE}_1\ne {PSE}_0 $$

and the test statistic PSE was

$$ PSE=\frac{{P\hat{S} E}_1\hbox{-} {P\hat{S} E}_0}{\sqrt{\mathrm{Var}\left({P\hat{S} E}_1\hbox{-} {P\hat{S} E}_0\right)}}. $$

The proposed PSE statistic was developed to test the difference of path-specific effects under two conditions.

### Non-parametric permutation and bootstrap tests of PSE

To test whether the specific path contributed to the disease end point, we conducted a series of hypothesis tests. The permutation-based hypothesis tests were conducted as follows: 1) draw a large number of data on disease status (e.g., case and control group) without replacement and estimate PSE in each group, and make difference between two groups and then forms our statistic PSE; 2) Repeat this process to form a permutation distribution under the condition *H*_0_ is true; 3) obtain the value of statistic actually observed in study and locate the value in permutation distribution to get the *P* value; 4) reject the null hypothesis (*H*_0_ : *PSE*_1_ = *PSE*_0_) if the *P* value is less than 0.05 [22]. While bootstrap tests were performed as follows: 1) draw a large number of bootstrap samples (e.g., 1000) and estimate PSE by two groups to form a bootstrap distribution; 2) define the 95% confidence interval (CI) of the bootstrap distribution; and 3) reject the null hypothesis (*H*_0_ : *PSE*_1_ = *PSE*_0_) if the CI does not include zero [23]. Three bootstrap interval methods were used including,

1. The Standard Norm Bootstrap Confidence Interval:

$$ \left(\hat{\theta}-{z}_{\alpha /2}{se}_B\left(\hat{\theta}\right),\kern0.9000001em \hat{\theta}+{z}_{\alpha /2}{se}_B\left(\hat{\theta}\right)\right) $$

2) The Basic Bootstrap Confidence Interval:

$$ \left({\hat{\theta}}_{\left[\left(B+1\right)\alpha /2\right]},\kern0.7em {\hat{\theta}}_{\left[\left(B+1\right)\left(1-\alpha /2\right)\right]}\right) $$

3) The Percentile Bootstrap Confidence Interval:

$$ \left(2\hat{\theta}-{\hat{\theta}}_{\left[\left(B+1\right)\left(1-\alpha /2\right)\right]}^{\ast },\kern0.5em 2\hat{\theta}-{\hat{\theta}}_{\left[\left(B+1\right)\left(\alpha /2\right)\right]}^{\ast}\right) $$

4) Bias correct confidence interval:

$$ \left({\hat{\theta}}_{\Phi \left(\mathrm{a}\right)}^{\ast },{\hat{\theta}}_{\Phi (b)}^{\ast}\right) $$

where$a=\left({\overset{\frown }{z}}_0+\frac{{\overset{\frown }{z}}_0+{z}_{\alpha /2}}{1-\overset{\frown }{a}\left({\overset{\frown }{z}}_0+{z}_{\alpha /2}\right)}\right),b=\Phi \left({\overset{\frown }{z}}_0+\frac{{\overset{\frown }{z}}_0+{z}_{1-\alpha /2}}{1-\overset{\frown }{a}\left({\overset{\frown }{z}}_0+{z}_{1-\alpha /2}\right)}\right)$. All codes for automatic searching specific paths linking two continuous variables and adjusting set as well as PSE statistic can be found in supplementary materials. 

### Simulation

Simulations were conducted to evaluate the performances of PSE statistic in the situation of varying across path-specific effect difference of *PSE*_1_ and *PSE*_0_ (e.g., Case group vs. Control group) and effects of parent nodes or child nodes on nodes on specific path as well as average causal effect value of every edge on specific path. We simulated a complex network on Myocardial Infarction and selected the target specific path: *Calorific excess* → *Visceral adiposit* → *Inflammatory z* ` *milieu* →  *Atherosclerosis* → *Myocardial infarction* (Fig. 2) to test our statistic. The simulation process was mainly based on their parent nodes to generate corresponding child nodes by logistic regression model. For instance, to generate the child node *Y* (*Visceral*) depends on corresponding parent nodes *X*_1_ (*Calorific*) and *X*_2_*(Physical inactivity*), log*it*(*P*(*Y* = 1| *X*_1_, *X*_2_)) = *α*_0_ + *β*_1_*X*_1_ + *β*_2_*X*_2_. Furthermore, we set different effects under two conditions on some specific paths and then identify the specific paths with different effects using PSE statistic.

Under the null hypothesis (*PSE*_*1*_ *= PSE*_*0*_), given the varied sample sizes (*N* = 200, 400, 600, 800, 1000), 1000 simulations were conducted to assess the type I error of the PSE by Permutation test and the non-parametric bootstrap tests with confidence interval (*CI*) estimated by Basic bootstrap, the percentile bootstrap and bias-corrected bootstrap methods and asymptotic normal distribution statistic *CI*. Under *H*_1_ (PSE_1_ ≠ PSE_0_). Given the sample sizes 1000, 1000 simulations were repeated to assess the power under varied path-specific effect difference (Case group vs. Control group) of specific path itself and their parent nodes or child nodes as well as average causal effect value of every edge on specific path, respectively.

Similarly, for continuous variales, according to the causal diagram in Fig. 6, we generated the simulated data via linear regression. We specified the differential directed edge X_2_ → Y in case and control designs and thus led to one differential specific path X_1_ → X_2_ → Y linking X_1_ to Y. The specific path effect in each group can be calculated as follows.

1. The specific path effect X_1_ → X_2_ → Y by adjusting for the parent node X_3_:

$$ \left[\frac{\partial }{X_1}E\left({X}_2|{X}_1\right)\right]\left[\frac{\partial }{X_2}E\left(Y|{X}_2,{X}_3\right)\right]. $$

(2) The specific path effect X_1_ → X_2_ → X_3_ → Y by separately adjusting for the parent nodes X_1_ and X_2_:

$$ \left[\frac{\partial }{X_1}E\left({X}_2|{X}_1\right)\right]\left[\frac{\partial }{X_2}E\left({X}_3|{X}_2,{X}_1\right)\right]\left[\frac{\partial }{X_3}E\left(Y|{X}_3,{X}_2\right)\right]. $$

(3) The specific path effect X_1_ → X_3_ → Y by separately adjusting for the parent nodes X_2_ and X_3_:

$$ \left[\frac{\partial }{X_1}E\left({X}_3|{X}_1,{X}_2\right)\right]\left[\frac{\partial }{X_3}E\left(Y|{X}_2,{X}_3\right)\right]. $$

Based on the pathway effects in two groups, we can develop PSE statistic via differenting the pathway effects in two groups.

All simulation codes were generated by R software available from CRAN (http://cran.r-project.org/).

## Supplementary information

**Additional file 1.** Codes for automatic calculating PSE statistic of all specific paths linking any two continuous variables.

## Data Availability

The data were downloaded from https://cancergenome.nih.gov/.

## Abbreviations

PSE: Path-specific effect
MTOR: Mammalian target of rapamycin
ACE: Average causal effect
WHO: The World Health Organization
GBM: Grade IV glioblastoma multiforme
