# Supplementary material for: A novel path-specific effect statistic for identifying the differential specific paths in systems epidemiology
Source: BMC Genet. 2020 Aug 8;21:85. doi: 10.1186/s12863-020-00876-w (PMC7414699; doi:10.1186/s12863-020-00876-w)
Supplement: Supplementary file 1 — Additional file 1. Codes for automatic calculating PSE statistic of all specific paths linking any two continuous variables. [file 12863_2020_876_MOESM1_ESM.pdf]

Codes for automatic calculating PSE statistic of all specific paths linking any two continuous variables

```
library(dagitty)
#########simulated data##########
N <- 10000 # sample size
D=rbinom(N,1,0.5)
Ux <- rnorm( N ); Uy <- rnorm( N ); Uz <- rnorm( N );Uw <- rnorm( N )
X <- Ux
Y <- (1/2+D)*X + Uy
Z <- (1/2+D)*Y + 1/2*X+Uz
W <- (1/2+D)*Z +1/2*X + 1/2*Y+Uw
d <- data.frame(X=X,Y=Y,Z=Z,W=W,D=D)
g <- dagitty("dag {
        Ux -> X -> Y -> Z <- Uz
        Uy -> Y
        X -> Z
        Z -> W
        X -> W
        Y -> W
        Uw -> W
}")
#coordinates(g) <- list(
  #      x=c(Ux=1,Uy=2,Uz=3,X=1,Y=2,Z=3),
    #    y=c(Ux=1,Uy=1,Uz=1,X=0,Y=0,Z=0) )
plot( graphLayout( g ) )

###########path-specific effect############
path_effect=function(d,g,start="X",end="W"){
P=paths( g, start, end, directed=TRUE )$paths    ##list the paths
###[1] "X -> Y -> Z"
N=length(P)
xy=rep(1,N)
for(j in 1:N){
    n=length(strsplit(P, split = " -> ")[[j]])##the number of paths
    for(i in 1:(n-1)){
        x=strsplit(P[j], split = " -> ")[[1]][i]
        y=strsplit(P[j], split = " -> ")[[1]][i+1]
        C= adjustmentSets( g, x, y, effect="direct" )[[1]]
        X1=d[,x]
        Y1=d[,y]
        if(length(C)==0){
            x_y=lm(Y1~X1,data=d)$coef[2]
        }else{
```

```
        C=d[,C]
      dd=cbind(X1,Y1,C)
      dd=data.frame(dd)
      x_y=lm(Y1~.,data=dd)$coef[2]
}
   xy[j] <- xy[j]*x_y
}
}
xy=matrix(xy,1,length(xy))
colnames(xy)=P
return(xy)
}

########################PSE statistic################

dif=function(d,vs,start="X",end="W",g){
##d is data
##vs is the colum for comparison
##g is the causal graph
d1=d[d[,vs]==1,]
d0=d[d[,vs]==0,]
d1=data.frame(d1)
d0=data.frame(d0)
effect1=path_effect(d1,g,start,end)
effect0=path_effect(d0,g,start,end)
dif=effect1-effect0
result=rbind(effect1,effect0,dif)
return(result)
}

###############main function####################
main=function(d,g,vs=5,start="X",end="W",permu=100){
##d is data
##g is the causal graph
##vs is the column of group variable
##The start and end are two target variables
##permu is the number of permutation test
diff.all=dif(d,vs=5,start,end,g)
diff=diff.all[3,]
len=length(diff)
pp=rep(0,len)
diff.per=matrix(0,permu,len)
for(i in 1:permu){
   index <- sample(d[,vs],length(d[,vs]),replace=FALSE)
```

```
    d[,vs]=index
    d2=data.frame(d)
    diff.per[i,]=dif(d2,vs=5,start,end,g)[3,]
}
pp=rep(0,len)
for(j in 1:len){
    pp[j]=(sum(abs(diff.per[,j])>=abs(diff[j])))/permu
}
result=rbind(diff.all,pp)
rownames(result)=c("D1","D0","D1-D0","p value")
return(result)
}

##############################Example###########
d <- data.frame(X=X,Y=Y,Z=Z,W=W,D=D) ##simulation data above
g <- dagitty("dag {
        Ux -> X -> Y -> Z <- Uz
        Uy -> Y
        X -> Z
        Z -> W
        X -> W
        Y -> W
        Uw -> W
}")
```

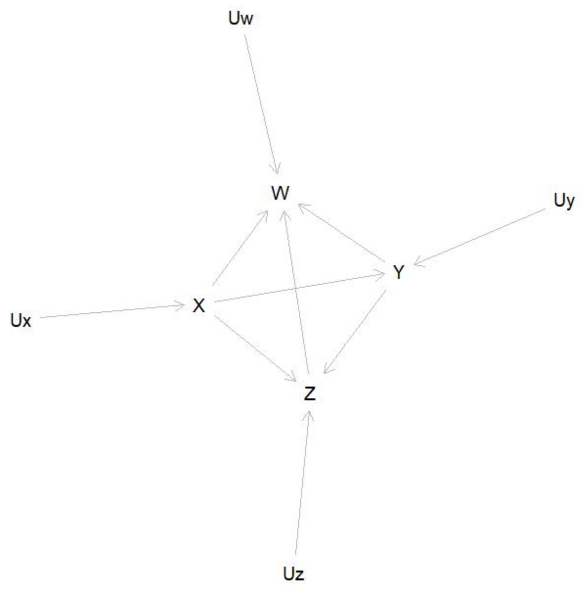

Figure 1 causal graph of g

```
vs=5

main(d,g,vs=5,start="X",end="W",permu=100)
                    X -> W          X -> Y -> W         X -> Y -> Z -> W      X -> Z -> W
```

D1	0.47326103	0.8011982	3.3073944	0.7627015
D0	0.50989327	0.2555094	0.1316245	0.2576758
D1-D0	-0.03663224	0.5456888	3.1757699	0.5050257
p value	0.37000000	0.0000000	0.0000000	0.0000000

##D1 is the PSE in group variable=1
##D0 is the PSE in group variable=0
##D1-D0 is the their difference
##p value is obtained by permutation test
